# Evidence of Reverse Zoonotic Transmission of Human Seasonal Influenza A Virus (H1N1, H3N2) Among Cats

**DOI:** 10.1111/irv.13296

**Published:** 2024-04-18

**Authors:** Sajid Umar, Semin Kim, Di Gao, Pu Chen

**Affiliations:** ^1^ Global Health Research Center (GHRC) Duke Kunshan University Kunshan China; ^2^ Division of Natural & Applied Sciences (DNAS) Duke Kunshan University Kunshan China; ^3^ MSD Animal Health Shanghai Shanghai China

**Keywords:** cats, China, influenza A virus, reverse zoonosis

Dear Editor,

Human–animal interactions are closely intertwined. The connection between animal, human, and environmental health is becoming increasingly complicated with globalization, industrialization, and climate change. Since the beginning of the 20th century, the number of domestic cats has increased rapidly worldwide, including in China. There are approximately 600 million domesticated cats worldwide, including 65 million cats in China, most of whom have close human contacts. These close contacts create more chances for pathogen spillover among humans and cats, which could lead to the emergence of new pathogenic strains or variants. Cats living in proximity to their owners carry a particular risk of catching pathogens, as they often share snuggles, kisses, dining, and beds [1, 2]. We share hundreds of pathogens with our animals, which they serve as intermediate or reservoir hosts for pathogens that affect human health. Cats, owing to their genetic similarity to humans, are more susceptible to catching diseases from their owners. Recently, it has been estimated that humans spillover far more pathogens to animals than animals transmit to humans [2, 3].

The natural transmission of disease and infection from humans (reservoir hosts) to animals is usually known as reverse zoonosis [4]. It is also called zooanthroponosis or anthroponosis and is considered the opposite of “zoonosis.” Reverse zoonotic events can form potential disease reservoirs that can reintroduce pathogens into human populations. Over the years, zoonotic events and pathogen spillover from animals to humans have been extensively studied, and reverse zoonotic events have remained understudied from animal health perspectives. Influenza viruses are genetically highly variable and remain the most significant concern for One Health. Influenza viruses now consist of four types within the family *Orthomyxoviridae*, including types A, B, C, and D. Type A and B influenza viruses are more abundant and are usually linked to seasonal flu outbreaks worldwide. Independent anthroponotic spillover of IAV viruses into farmed, captive, and wild animals has been reported [5, 6].

The high susceptibility of cats to IAVs generates the possibility of zoonotic and reverse‐zoonotic transmission events and can serve as a mixing vessel for the emergence of new IAV variants (Figure 1). A large population of animals and humans makes China an ideal location for the emergence of future IAV pandemics.

**FIGURE 1:**
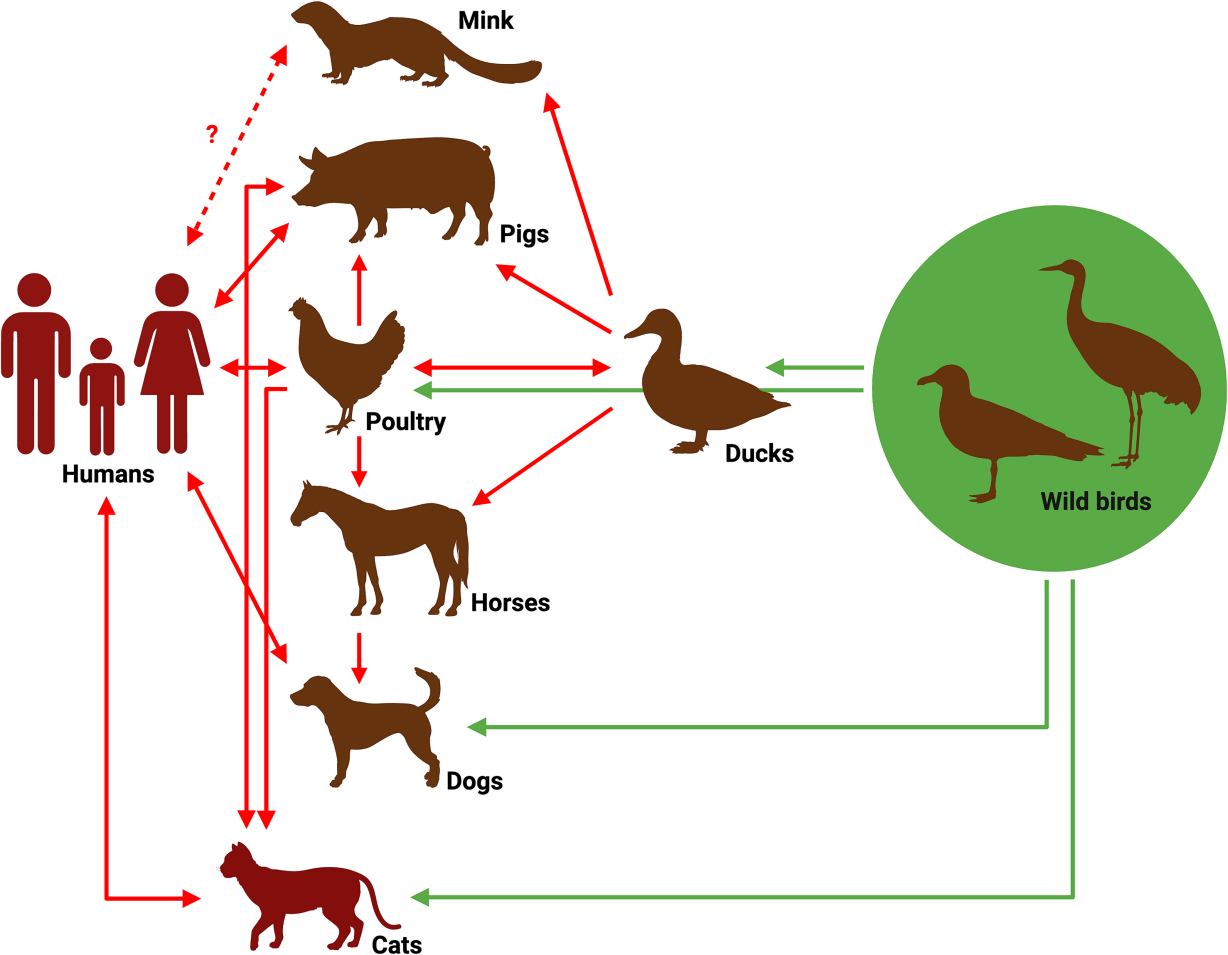
Zoonotic and reverse zoonotic events of influenza A viruses among different animal species (adapted from Kibenge [4] with modifications, www.biorender.com 2024).

There is a scarcity of data on the reverse zoonosis of human seasonal IAV strains (H1N1, H3N2) in China, especially in cats. For continuous monitoring of reverse zoonotic transmission and evolution of human influenza viruses in China, we collected 458 nasal swab samples from diseased cats exhibiting clinical signs of respiratory infection from veterinary clinics in Kunshan City, Jiangsu Province, China, during 2021–2024. After collection, nasal swabs were submerged in a vial containing sterile virus transport media (Copan Diagnostics Inc., Italy) and processed for RNA extraction using Takara Minibest viral RNA extraction kits (Cat#9766, Takara, Dalian, China). RNA samples were screened for influenza viruses by real‐time RT‐PCR (RT‐qPCR) using an MIC Real Time qPCR cycler (Biomolecular Systems, Australia) with a Superscript III One‐Step RT‐PCR System with Platinum Taq polymerase (Thermo Fisher Scientific Inc.). Human seasonal IAV strains (H1N1 and H3N2) and influenza B viruses (B/Yamagata or B/Victoria) were detected by following the protocols described previously [7]. Positive and negative controls were added to each reaction to optimize and validate the assays. Samples that tested positive by RT‐qPCR were further tested by hemagglutination inhibition assay (HAI) to screen for the presence of antibodies against IAV (H1N1 and H3N2).

IAV was detected in 2.8% of the samples (13/458), whereas influenza B virus was not detected during this study. Genetic analysis revealed the presence of A (H1N1) and A (H3N2). Among the positive strains, there were nine strains of A (H1N1) virus and four strains of A (H3N2) virus. A/H1N1 and A/H3N2 positive cats showed HAI titers against these viruses, which also supported the evidence of reverse zoonosis. Interestingly, a higher detection rate (84.61%) was observed in samples collected during autumn and winter, which could be linked to the peak flu season in Kunshan and Shanghai. Clinical signs, including sneezing, dyspnea, and coughing, varied from mild to moderate among influenza‐positive cats. No deaths were reported among the positive cats. Based on molecular and serological testing, we demonstrated human seasonal IAV‐infected cats in this study. This is the first report to assess the reverse zoonotic events of influenza viruses in cats in Kunshan, China, and highlights the potential risk of catching IAV in cats living in close contact with their owners. Despite some limitations, such as the small sample size and geographical area, our study provides useful information to veterinarians, pet owners, and policymakers.

Cats could act as additional intermediate or reservoir hosts for endemic IVA evolution and thus may contribute to major public health issues. There are several reports on the natural transmission of different IAV subtypes in cats, including avian H5N1, canine H3N2, human H1N1, and H3N2 [8]. Anthroponotic spillover events for IAV have been documented among cats, suggesting close interactions between cats and owners suffering from influenza‐like illness [1, 8, 9, 10, 11]. In addition to cats, a variety of other animals (swine, dogs, turkeys, and ferrets) has been naturally infected with Influenza A/H1N1. Human‐to‐pig transmission of IAV is the most studied anthroponotic event. More IAVs jump from humans to swine than from swine to humans [1]. Several suggestions have been made to minimize the risk of IAV‐reverse zoonosis. First, flu vaccine shots are recommended to owners and susceptible cats to reduce anthroponotic events. Second, people who are sick with seasonal flu need to modify their behaviors and should be more vigilant about the health of their cats. Pet owners can minimize reverse zoonotic transmission by keeping nasal discharges and other bodily secretions away from cats during the sickness period. Third, owners can minimize their playing time and petting activities with their cats while they are sick. Finally, they can also limit reverse zoonotic transmission by regularly cleaning and disinfecting the bedding area and providing fresh and healthy feed to their cats.

Reverse zoonotic events in IAV can pose significant health risks for cats and possibly human health if left unchecked. Therefore, it is important to keep the IAV under control before it imposes deadly consequences on the human population. Keeping in mind the close association of cats with humans and the high pandemic potential of IAV warrants a more integrated research approach to minimize reverse zoonoses. This could include greater testing and continuous human pathogen surveillance at the human‐animal contact interface. This type of data could facilitate the mitigation, prevention, prediction, and preparation of future IAV pandemics.

## Author Contributions

**Sajid Umar:** Conceptualization; Investigation; Funding acquisition; Writing – original draft; Methodology; Validation; Visualization; Writing – review and editing; Formal analysis; Data curation; Supervision; Resources. **Semin Kim:** Writing – original draft; Writing – review and editing. **Di Gao:** Conceptualization; Writing – review and editing. **Pu Chen:** Conceptualization; Writing – review and editing.

## Conflicts of Interest

The authors declare no conflicts of interest.

### Peer Review

The peer review history for this article is available at https://www.webofscience.com/api/gateway/wos/peer‐review/10.1111/irv.13296.

## Data Availability

The data that support the findings of this study are available on request from the corresponding author. The data are not publicly available because of privacy or ethical restrictions.
